# Knowledge and Attitudes of Obstetrics and Gynecology and Family Medicine Residents Regarding Transgender and Gender-Diverse Health: A Multicenter Cross-Sectional Study

**DOI:** 10.3390/healthcare13131596

**Published:** 2025-07-03

**Authors:** Ozlem Ozgun Uyaniklar, Hikmet Altun, Yesim Uncu

**Affiliations:** 1 Department of Obstetrics and Gynecology, Bursa City Hospital, Nilufer 16110, Bursa, Turkey; dr.hikmetaltun@gmail.com; 2 Department of Family Medicine, Uludag University Faculty of Medicine, Nilufer 16059, Bursa, Turkey; yuncu@uludag.edu.tr

**Keywords:** transgender and gender diverse health, primary healthcare, healthcare disparities, graduate medical education

## Abstract

**Objectives**: Health disparities among transgender and gender-diverse individuals are partly attributed to the limited knowledge and preparedness of healthcare providers. This study aims to assess the level of knowledge of transgender health among residents in obstetrics and gynecology and family medicine. **Methods**: A multicenter, cross-sectional study was conducted with obstetrics and gynecology (OBGYN) and family medicine (FM) residents from two different centers. Data were collected using a 21-item questionnaire. The questionnaire assessed participants’ demographic information, knowledge of sexual orientation and gender identity, clinical and anatomical effects of gender-affirming hormone therapy, knowledge of gender-affirming surgery, and knowledge of risk-based screening for TGD individuals. **Results**: The study, which included 85 residents (62 FM, 23 OBGYN), found that 76.6% of respondents indicated that SOGI should be routinely assessed. However, 68.3% of the participants reported inadequate knowledge regarding the clinical implications of gender-affirming hormone therapy, and 74.1% reported insufficient knowledge about gender-affirming surgeries. Furthermore, 62.4% of the participants indicated that they had not received any health education specifically tailored to address the needs of transgender individuals. Only 23.5% reported receiving training during their residency programs. Notably, 84.7% of the participants expressed a desire for formal education in this area. **Conclusions**: FM and OBGYN residents have significant knowledge gaps regarding TGD health. The integration of TGD health topics into the curricula of medical schools and residency programs is needed to improve access to healthcare for individuals with TGD.

## 1. Introduction

The term “transgender” is often used to describe people whose gender identity or expression is different from the sex they were assigned at birth [1]. Gender diversity is defined as a range of gender identities and expressions that do not conform to traditional expectations of masculinity and femininity imposed by society or culture [2]. In this text, the term TGD specifically refers to transgender and gender-diverse individuals across the spectrum of sexual orientations and gender identities [1]. Gender identity is a deeply personal experience, and the ways transgender individuals express and affirm their identity can vary greatly depending on cultural, social, and individual contexts [1,2]. Therefore, it is crucial to understand the diversity observed among transgender individuals in order to provide respectful, informed, and inclusive care and support [2].

TGD individuals are frequently subjected to various forms of prejudice, including harassment, discrimination, and social rejection [3]. They often encounter discriminatory attitudes and behaviors in healthcare settings, particularly concerning access to qualified medical professionals who are knowledgeable about transgender medicine [3,4]. The necessity for special considerations in primary and preventive care may vary depending on an individual’s history of gender-affirming care. TGD individuals frequently face significant barriers to accessing healthcare, including financial limitations, discrimination, inadequate cultural competence among providers, systemic obstacles, and socioeconomic challenges [5]. These barriers contribute to the underutilization of healthcare services among TGD individuals and often lead them to delay seeking professional medical care until acute problems arise [4]. Health disparities among transgender individuals, particularly regarding mental health, are often explained by minority stress theory [6]. This theory states that stigmatized groups experience chronic, identity-related stress due to societal non-affirmation, including in legal and healthcare systems [6]. The poorer health outcomes observed in TGD populations are often exacerbated by systemic discrimination and social stigma [7].

One of the major barriers to equitable healthcare for TGD people is the limited access to healthcare providers who are knowledgeable about TGD health [7,8]. Healthcare providers’ discomfort in interacting with TGD individuals, along with a perceived lack of preparedness to deliver appropriate care, may significantly contribute to these disparities [4]. Many professionals report limited opportunities to receive training on TGD health during their medical education. This reflects a broader issue: conventional medical curricula often lack sufficient content related to TGD health, resulting in significant knowledge gaps among physicians and potentially leading to inadequate care for TGD populations [9,10,11]. This curricular gap highlights a structural barrier in medical education systems. It aligns with the concept of structural competency, which emphasizes understanding how institutional practices and systemic factors shape healthcare disparities.

Family physicians and OBGYNs are among the first points of contact for TGD individuals within the healthcare system. It is essential that relevant healthcare professionals possess sufficient knowledge and awareness of TGD health to ensure the provision of inclusive healthcare services [3,12]. Adequate training in this area has been shown to improve clinical approaches to issues such as gender-affirming care, reproductive health services, and access to screening programs [13,14]. Furthermore, it plays a key role in addressing issues of structural discrimination and bias within healthcare services [15,16,17].

To address the gaps in residency training, we identified specific areas of deficiency and accordingly designed this study. A comprehensive literature review was conducted using databases such as PubMed, Scopus and Web of Science along with keywords including “transgender health education,” “residency training,” “family physicians,” and “OB-GYN.” The search revealed a limited number of studies specifically addressing structured educational needs in this context, particularly within developing countries. This gap emphasizes the necessity and originality of the current study.

The primary aim of this study is to evaluate the education on TGD health provided during medical and residency training. Through a survey involving residents in Family Medicine (FM) and Obstetrics and Gynecology (OBGYN), the study plans to assess the knowledge level regarding transgender health-related issues.

## 2. Materials and Methods

### 2.1. Study Popılation and Data Collection

This study was a multi-center, cross-sectional study that was conducted from December 2023 to December 2024 by including residents of OBGYN and FM residency programs in the province of Bursa from two different centers. To maintain consistency in educational background and training curricula, this research was limited to a single province. Expanding the study to a broader geographic area would have necessitated the use of an online survey format; however, this approach was deliberately avoided due to several limitations commonly associated with online data collection. These include a higher likelihood of superficial responses, lower participation rates—since individuals often ignore or delete survey emails—and potential barriers for participants who are not technologically proficient. The participants were given detailed explanations about the purpose and process of the research, and they were invited to participate voluntarily. Written informed consent was obtained from all participants before the study.

This study included resident physicians working in OBGYN and FM clinics. The inclusion criteria were as follows: being a resident in one of these specialties, having completed at least one year of residency training, not yet having obtained a specialist title, being actively engaged in clinical training, and voluntarily agreeing to participate in the survey. Exclusion criteria consisted of residents who had transferred from other specialties (e.g., internal medicine, pediatrics) into OBGYN or FM and those with less than one year of residency experience. Residents on rotation in other departments, those on leave, or those not actively practicing clinically were excluded to ensure a more representative sample.

Data were collected through a survey administered to the participants. The questionnaires were distributed in person during departmental meetings, and participants completed and returned them on site. The data collection process did not involve the utilization of online tools or digital platforms. The survey aimed to assess the knowledge of FM and OBGYN physicians regarding the healthcare of TGD individuals, with a focus on content relevant to both specialties. The authors conducted a comprehensive review of the current literature on TGD health and developed a 21-item questionnaire based on the identified knowledge domains. The survey was pilot-tested with academics from the fields of FM and OBGYN to evaluate the clarity, relevance, and comprehensiveness of the items. This was necessary because a validated instrument on this subject was not available. Responses were measured using a binary Likert scale (“disagree” as a negative response; “agree” as a positive response). Demographic data were also collected via a form provided alongside the survey. All data were collected anonymously. Although the use of a dichotomous scale may limit the ability to capture nuanced attitudes and levels of knowledge, it is a methodologically acceptable choice in exploratory research—particularly when addressing emerging and understudied topics such as transgender health. In such contexts, a two-option format allows for the collection of clear and straightforward responses, especially when participants’ attitudes or knowledge frameworks are not yet well-established in the literature.

The analysis included participants’ age, gender, marital status, years of residency, and area of specialty. The survey comprised 21 questions, starting with Questions 1 and 2, which focused on sexual orientation and gender identity (SOGI). Question 3 assessed knowledge about sexual anatomy, development, and behavior. Questions 4 and 5 addressed the clinical and anatomical implications of gender-affirming hormone therapy, while question 6 addressed participants’ understanding of gender-affirming surgery. Questions 7 through 14 evaluated knowledge regarding risk-based screening for TGD individuals, covering aspects like hormone therapy, cancer screenings, and screenings for sexually transmitted infections (STIs). Question 15 gauged awareness of the heightened risk of discrimination and hate crimes faced by TGD individuals, and Question 16 examined barriers to accessing and utilizing healthcare services. Questions 17 through 20 investigated whether participants had received formal education on TGD health, when it occurred during their training, and if they deemed it adequate. Lastly, Question 21 inquired if participants were interested in further education on TGD health.

### 2.2. Ethics Committee Approval

The study protocol was approved by the Uludağ University Faculty of Medicine Clinical Research Ethics Committee at the beginning of the study (approval number 2023-26/4, approval date: 7 December 2023).

### 2.3. Statistical Analysis

Statistical analysis was performed using SPSS version 23 (IBM Inc., Armonk, NY, USA). Normality was assessed with the Kolmogorov–Smirnov test. The Mann–Whitney U test was used to compare two independent categorical variables for non-normally distributed data. Associations between categorical variables were examined using Fisher’s Exact test. Binary logistic regression analysis was applied to assess factors influencing the dependent variable. Quantitative data are presented as mean ± standard deviation and median (minimum–maximum) values. Categorical data are presented as frequencies and percentages. The logistic regression analysis results are reported as odds ratios (ORs) with 95% CIs; *p* < 0.05 was considered statistically significant. The internal consistency of the questionnaire was assessed using Cronbach’s alpha coefficient. For the overall reliability, the Cronbach’s alpha value was 0.850.

## 3. Results

Residents from two residency programs participated in this study. At the outset, there were 105 residents across the FM and OBGYN clinics. Of these, seven first-year residents and one resident who had transferred from another specialty were excluded based on study criteria. Among the eligible residents, seven were excluded due to incomplete questionnaires, and five declined to participate. Consequently, a total of 85 residents were included in the final analysis, comprising 62 from the FM clinic and 23 from the OBGYN clinic. The demographic characteristics and survey results are detailed in Table 1.

There was no significant difference in the duration of residency between the two clinics. However, the median age was 30 years in the FM group and 28 years in the OBGYN group, showing a significant difference (*p* = 0.009). When evaluating the survey responses, 76.6% of participants believe that SOGI should be routinely assessed (Question 1). Regarding terminology related to these topics, 87.1% of respondents felt they had sufficient medical knowledge. However, 68.3% reported lacking knowledge about the clinical effects of gender-affirming hormone therapy, and 73% indicated inadequate understanding of its anatomical effects (Questions 4 and 5).

Additionally, 74.1% of participants stated they do not have enough knowledge about gender-affirming surgeries (Question 6). In terms of counseling for Pap test screening for cervical cancer in appropriate TGD patient groups, 65.9% reported insufficient knowledge (Question 8). Conversely, more than 50% of participants felt they had adequate knowledge regarding risk-based healthcare counseling for TGD populations, including screening for STIs, prostate cancer, mammography, osteoporosis, and hormone therapy, particularly estrogen use (Questions 7, 9–14).

A significant 90.6% of participants acknowledged that TGD individuals often face discrimination and hate crimes, which may contribute to a higher incidence of psychiatric disorders (Question 15). When asked if they had received formal medical training on assessing SOGI, 54.1% of participants indicated they had not received such education. Furthermore, 62.4% reported that they had never undergone any formal training related to TGD health (Questions 17 and 18). In response to the question about the timing of training on TGD health, 12.9% of participants reported training during medical school and 23.5% during residency (Question 19). Additionally, 89.4% of respondents stated they had not received sufficient training (Question 20), while 84.7% wished to learn more about this subject (Question 21). 

A numerical difference existed between the number of participants from each department: FM (n = 62) and OBGYN (n = 23). Therefore, regression analysis was conducted to assess the impact of potential confounders—such as age, gender, department, and years in training—on the survey responses. The findings from this analysis are detailed in Table 2. Notable differences were found in the regression analysis results for two specific questions. The univariate analysis revealed that being part of the OBGYN group significantly heightens the risk of having insufficient knowledge about counseling for STIs compared to the FM group (Question 13) (OR: 4.024, 95% CI: 1.092–14.831, *p* = 0.036). This relationship remained significant in the multiple regression analysis (OR: 4.876, 95% CI: 1.146–20.747, *p* = 0.032), indicating that even after accounting for other variables, the department affiliation significantly impacts knowledge levels regarding STI screening in TGD individuals. In the univariate analysis, a longer duration of assistantship was significantly linked to a lower risk of lacking formal education (Question 18) (OR: 0.57, 95% CI: 0.34–0.957, *p* = 0.033). This association continued to be significant in the multiple regression analysis (OR: 0.576, 95% CI: 0.334–0.993, *p* = 0.047)

## 4. Discussion

This study is of critical importance as it systematically identifies gaps in TGD healthcare knowledge among OBGYN and FM resident physicians, with the aim of informing targeted educational improvements. The study uses structured survey questions to assess key areas such as sexual orientation/gender identity terminology, anatomy, gender-affirming therapies (hormonal/surgical), risk-based screenings, and barriers to care, such as discrimination and healthcare access disparities.

The study’s findings will spotlight urgent educational shortcomings. They will also provide actionable evidence to guide mandatory curriculum reforms. The study identifies specific knowledge gaps that can serve as a roadmap for medical educators in designing targeted educational interventions. They can use the roadmap to redesign training modules. They can also integrate standardized TGD health competencies.

The study was conducted among residents in FM and OBGYN specialties, and only 10.6% felt they received adequate training (question 20); this underlines how important it is to make further, more significant improvements to both curricula of residency and medical school in teaching the TGD healthcare subject.

Family physicians play a very significant role in the health of TGD individuals, as they provide primary healthcare services. OBGYNs also play an extremely important role in the routine care of these patients, and specific education and clinical skills training are required to address the unique healthcare needs of TGD individuals. We therefore included residents from these two specialties in our study.

One of the most impressive results of our research was question 18, which asked if they had ever received any training on healthcare for TGD individuals; 62.4% of the participants reported that they had not received any training at all. Multiple regression analyses show that only the length of years in residency has a statistical significance in this question. Both medical school and residency curricula are deficient in TGD health, which unfortunately contributes to the health disparities experienced by TGD individuals [18]. Inadequate training and education of health professionals in transgender medicine may be associated with problems in the doctor–patient relationship, discriminatory attitudes, and lack of medical knowledge about TGD health [15,16,17]. A study by Obedin-Maliver et al. found that the medical students spent a median of 5 h discussing TGD health issues during the combined preclinical and clinical year of medical school; 33.3% reported no hours during the clinical year [18]. In our study, the response rate of participants who reported receiving training about TGD healthcare during medical school education was 12.9%; this supports the data in the literature. Most of the studies in the current research on this topic have reported a low level of comfort and knowledge among medical students [19].

In a study conducted by Unger et al. with OBGYN specialists, it was reported that 80% of the participants responded that they had not received any training in transgender care during their residency training [11]. A cross-sectional survey of OBGYN residency programs found that 50% of residency programs offer training in TGD health. Directors of non-providing programs cited lack of faculty experience, lack of curriculum, and lack of funding as reasons [20]. However, an analysis of the data yielded by our study indicates that the rate of education during the residency program is considerably lower.

According to the American College of Obstetricians and Gynecologists, in a Committee Opinion, OBGYNs should provide guidance on routine screening and transitional care. They have an identified need for understanding the nuances regarding TGD people and preparation to provide preventive health maintenance and being knowledgeable about hormone treatments and surgeries. In the 13th edition of the Council on Resident Education in Obstetrics and Gynecology (CREOG) Educational Objectives, TGD health is addressed in several contexts [21]. According to a study by Grimstad et al., 72% of directors of OBGYN residency programs were knowledgeable about the TGD health content of the prior version of CREOG educational objectives, that of 2013 [22]. As a result of this study, awareness is inconsistent across all regions and types of programs. University-based program directors expressed the highest familiarity level, 82.5%. Most programs utilize educational lectures at 62.9% and reading material at 51.6%. These methods are by no means adequate, while 14.5% subjects did not use educational material at all [22].

Family physicians are in a position to care for TGD patients, but few are trained in this care during residency training [23]. Among FM residency program directors, in a survey, 57.9% of the respondents reported currently incorporating TGD healthcare-related topics into the FM residency curriculum [23]. A study by Coutin A et al. showed that while 71% of FM residents felt that TGD health issues were within their area of expertise, only 10% felt competent to provide TGD healthcare at the end of residency [12]. In a study that sought to assess the current state of comprehensive healthcare education for TGD people in FM residency programs within the United States and Canada, data were collected via an online survey of FM residency program directors [24]. Where 66% of the program directors believed that more education on this subject should be provided in their institutions, 77% of the programs did not have TGD healthcare education.

Notably, age and gender distribution were significantly different between the FM and OBGYN groups in our study; however, the duration of residency, which may also affect the outcome of the study, was insignificantly different between the two groups.

Evidence from various studies suggests that the greater the clinicians’ knowledge of SOGI, and awareness of questioning, the better the positive health outcomes among TGD patients [13]. In our study, 76.6% of participants responded positively to the question about the need to ask about SOGI demographics (Question 1). Ensuring regular collection of structured patient data on SOGI in electronic health records (EHRs) is proposed as a key tool to identify, address, and ultimately reduce TGD health disparities [25]. In a study involving 10 health centers serving 441,387 patients, a project was developed to increase the capacity of healthcare provision for TGD people [14]. Health workers received coaching on creating TGD-inclusive environments, collecting SOGI data, conducting risk-based screening, and asking about sexual history. After 1 year, an evaluation showed that SOGI documentation in EHRs increased from 13.5% to 50.8% and risk-based screening [14]. In our study, regarding how to inquire about SOGI (Question 17), 54.1% of the participants reported that they had not received any training on this topic.

TGD individuals have unique healthcare needs in addition to the routine care, including primary and secondary prevention [26]. Gender affirmation is a multidisciplinary treatment, so not all primary care clinicians will be able to offer all elements of comprehensive care, but every clinician can become comfortable working with TGD patients to meet their health needs, including gender-affirming interventions. Hormone therapy can be managed either by a specialist or a primary care provider [27]. Physicians who are not directly involved in the management of hormone therapy should be aware of commonly prescribed hormone medications, their typical doses, potential side effects, and when to refer patients to specialists. In our survey, 31% of the participants indicated that they had sufficient knowledge about the clinical effects of gender-affirming hormone therapy, while 27.1% reported sufficient knowledge about its anatomical effects. Only 25.9% of the participants reported having sufficient knowledge about gender-affirming surgery. A detailed history of past and current use of gender-related hormones and gender-affirming surgery is very important. It should be known that hormone therapy can be medically supervised or unsupervised [28]. In a cohort study conducted at a university gender clinic, 1361 transgender patients were examined for mortality after at least one year of hormonal use and a median follow-up of 18.5 years [29]. Male-to-female (MtF) individuals on long-term cross-gender hormone therapy had a 51% higher mortality rate than the general population, primarily due to non-hormone-related causes such as suicide, AIDS, and cardiovascular disease. However, current use of ethinyl estradiol was associated with a threefold increased risk of cardiovascular death [29].

In our study, 69.4% of the participants responded positively to the question “There should be risk-based screening related to SOGI” (Question 7). Screening and prevention of chronic diseases has some special considerations for TGD patients compared to the general population. There is evidence that the use of feminizing hormones may have adverse effects on lipid profile and insulin sensitivity, increasing the risk of cardiovascular disease [30]. When asked about the cardiovascular risks of estrogen use, 67% of the participants in our study responded positively that they could provide counseling.

Women who have sex with women (WSW) often skip gynecological check-ups, believing they do not need contraception and that their risk of sexually transmitted infections is low [31]. In our study, there were two questions related to Pap smear testing. The first (Question 8) assessed adequate knowledge about counseling TGD patients on Pap smear testing. For this question, 65.9% of participants provided a negative response. On the other hand, 88.2% of participants gave a positive response to the question about Pap smear testing for WSW (Question 9). When all TGD individuals are included in the assessment, an even greater need for knowledge becomes apparent. This likely reflects gaps in understanding gender-affirming anatomy and terminology—for example, recognizing which patients have undergone gender-affirming surgery or distinguishing between total and subtotal hysterectomy. In contrast, participants demonstrated higher knowledge levels when recommending Pap tests to WSW, probably because this represents a more clearly defined and familiar screening category. No significant differences were observed for either question concerning factors such as specialty fields or duration of residency. According to one study, 25% of WSW postponed cervical cancer screening due to fear and discrimination [32]. WSW have a higher risk of cervical cancer than heterosexual women due to less screening [32]. Regarding cancer screenings, 41.1% and 49.4% of the participants reported inadequate knowledge about prostate cancer screening and breast cancer screening, respectively. The history of gender-affirming surgery and hormone therapy are important in this regard. It seems that there may be an increased risk of breast cancer with a longer length of exposure to feminizing hormones [33]. Cancer screening and treatment are not altered by SOGI, but there are many negative factors in these patients’ access to screening and treatment. Problems with health insurance, negative experiences with health services, and patients thinking that ‘screening is not necessary’ can be listed as some of the negative factors [34]. The potential for decreased access to healthcare services may contribute to the observed lower rates of screening for cervical cancer and increased risk of delayed diagnosis of breast cancer among TGD Individuals [35,36].

Sexually transmitted infections (STIs) are infections caused by pathogens that spread primarily through sexual contact [37]. Primary prevention of STIs involves assessing sexual behaviors that put people at risk of infection and assessing biological risk. Healthcare providers should routinely obtain sexual histories and discuss risk reduction as part of the clinical encounter [37]. In our study, 87% of the participants responded positively to the question about the availability of adequate information on STIs and screening methods (Question 13). After performing the regression analysis, the adjusted model revealed that being in the OBGYN department was significantly associated with inadequate knowledge regarding routine follow-up and screening for TGD individuals, with the odds ratio 4.876 (95% CI: 1.146–20.747; *p* = 0.032). Family physicians assess the health risks of the community they are responsible for, provide preventive health services, perform early diagnosis and follow-up of diseases, manage chronic diseases and refer individuals to specialized health services when necessary. We attribute the significant difference in the level of knowledge on risk-based screening for STIs to the primary role of FM Specialists in the health system in this risk-based screening. During specialty training, they receive training on screening, prevention, and treatment of ‘infectious diseases’, albeit under different titles within the curriculum.

When we asked the participants if they would like to receive medical training on TGD health, 84.7% said they would like to receive it. This is actually the most important point of support for the steps that can be taken on this issue. We suggest adding several practical, student-friendly elements to existing residency programs. First, three to four short, focused sessions on TGD health that cover terminology, patient interviews, and psychosocial support could be added. To reinforce practical skills, anonymized case-based small group discussions and standardized patient simulations could be used to strengthen both communication competencies and clinical decision-making. Additionally, flexible-access online e-learning modules and webinars could increase student engagement. Finally, pairing each trainee with an experienced clinician mentor for one-on-one feedback could help them translate new knowledge into daily practice and support the long-term sustainability of these educational interventions.

Our study reveals significant gaps in the existing literature, especially regarding residency training, and addresses these critical deficiencies. Our study shows that current residency programs lack standardized education on TGD healthcare. Using a structured, original survey that encompasses diverse domains—from sexual anatomy to barriers to accessing healthcare—our study maps existing knowledge and training deficiencies. Furthermore, our study provides field-specific data to assist educational accreditation committees in ensuring core competencies in TGD healthcare.

### Limitations

Our study has some limitations. First, the study was conducted in a single province in Turkey, which may limit the generalizability of our outcomes. It is important to note that our results may not fully apply to residents training elsewhere. Another potential limitation is that we used a self-developed, non-validated questionnaire that demonstrated acceptable internal consistency (Cronbach’s α = 0.850). We also relied on self-reported knowledge rather than direct assessments of clinical skills, meaning there may be a difference between reported competence and actual competence. While the majority of residents expressed their willingness to participate, five individuals chose not to take part, which may introduce a potential selection bias. Finally, we used a two-point Likert scale (agree/disagree) to keep the survey as straightforward as possible. However, it is possible that the structure of the scale may have restricted the participants*’* capacity to articulate more nuanced perspectives. Future research may benefit from employing more nuanced response formats, such as open-ended questions or multi-point Likert scales (e.g., 5-point or 7-point), which allow for more detailed statistical analysis and a deeper understanding of participants’ knowledge and attitudes.

## 5. Conclusions

The results of our study show that physician residents in the branches active in providing healthcare to TGD people have a very high level of inadequate knowledge about TGD health. Identifying critical knowledge gaps in TGD health will help ensure that future healthcare providers are competent in addressing TGD-specific health needs by customizing specialized training programs. Additionally, highlighting systemic barriers (e.g., discrimination, inadequate screenings) that assistants need to learn to address will promote equitable healthcare delivery, and providing evidence-based data to advocate for a standardized healthcare curriculum will increase assistants’ clinical confidence and TGD patients’ access to healthcare. Based on the results of this study, we plan to pilot changes in residency training programs to include TGD healthcare in the curriculum. Therefore, the evaluation and examination of TGD individuals by physicians who are specifically trained in this field have a positive impact on their health and well-being. Our study highlights the lack of education on transgender health and emphasizes the importance of increasing education in both medical school and specialty curricula. And we aim to be a pioneer in this field to address this gap in the curricula of residency programs, including medical school curricula.

To build on these efforts, future studies—preferably conducted on a larger scale—should evaluate the long-term impact of curricular changes on residents’ clinical competence in TGD healthcare, and also assess systemic barriers (e.g., institutional policies, insurance coverage) that may hinder their ability to practice gender-affirming care effectively.

## Figures and Tables

**Table 1:** Demographic characteristics and survey responses of participants.

	Family Medicine (n = 62)	Obstetrics and Gynecology (n = 23)	*p*
Age	30 (25–59) 31.61 ± 6.29	28 (24–35) 28.22 ± 2.49	0.009 ^a^
Assistantship duration	2 (1–5) 1.79 ± 1.01	2 (1–2) 1.52 ± 0.51	0.584 ^a^
Gender			0.002 ^b^
Female	34 (54.8)	19 (82.6)	
Male	27 (43.5)	2 (8.7)	
Prefer not to respond	1 (1.6)	2 (8.7)	
Marital Status			0.102 ^b^
Married	34 (54.8)	7 (30.4)	
Single	27 (43.5)	15 (65.2)	
Other	1 (1.6)	1 (4.3)	
**Survey Results**			
	**Response**	**Frequency**	**Percentage**
Q1	Negative Response	20	23.6
	Positive Response	65	76.6
Q2	Negative Response	11	13
	Positive Response	74	87.1
Q3	Negative Response	6	7.1
	Positive Response	79	92.9
Q4	Negative Response	58	68.3
	Positive Response	27	31.8
Q5	Negative Response	62	73
	Positive Response	23	27.1
Q6	Negative Response	63	74.1
	Positive Response	22	25.9
Q7	Negative Response	26	30.6
	Positive Response	59	69.4
Q8	Negative Response	56	65.9
	Positive Response	29	34.1
Q9	Negative Response	10	11.8
	Positive Response	75	88.2
Q10	Negative Response	35	41.1
	Positive Response	50	58.8
Q11	Negative Response	42	49.4
	Positive Response	43	50.7
Q12	Negative Response	28	32.9
	Positive Response	57	67
Q13	Negative Response	11	13
	Positive Response	74	87
Q14	Negative Response	14	16.5
	Positive Response	71	83.5
Q15	Negative Response	8	9.4
	Positive Response	77	90.6
Q16	Negative Response	21	24.7
	Positive Response	64	75.3
Q17	Negative Response	46	54.1
	Positive Response	39	45.9
Q18	Negative Response	53	62.4
	Positive Response	32	37.6
Q19	Medical School Education	11	12.9
	Specialty Education	20	23.5
	Other	0	0
	No Education Received	54	63.5
Q20	Negative Response	9	10.6
	Positive Response	76	89.4
Q21	Negative Response	13	15.3
	Positive Response	72	84.7

^a^: Mann–Whitney U test; ^b^: Fisher’s Exact Test. Data are presented as mean ± SD and median (min-max) for normally distributed and skewed quantitative variables, respectively; and n (%) for nominal variables.

**Table 2:** Assessment of the effect of independent variables on survey responses using binary logistic regression analysis.

	Univariate OR (%95 CI)	*p*	Multiple OR (%95 CI)	*p*
**Inadequate knowledge level regarding sexual orientation and gender identity terminology (Q-2)**				
Age	0.963 (0.841–1.103)	0.586	0.944 (0.76–1.172)	0.603
Gender (Reference: Female)	0.487 (0.094–2.514)	0.39	0.763 (0.12–4.861)	0.775
Department (Reference: Family Medicine)	4.024 (1.092–14.831)	**0.036**	2.439 (0.494–12.051)	0.274
Assistantship duration	1.142 (0.591–2.207)	0.693	1.599 (0.75–3.408)	0.225
**Inadequate knowledge of sexual anatomy, sexual development, and behavior related to TGD individuals (Q-3)**				
Age	1.005 (0.871–1.158)	0.95	0.998 (0.777–1.281)	0.988
Gender (Reference: Female)	0.595 (0.059–5.997)	0.66	0.892 (0.061–13.026)	0.933
Department (Reference: Family Medicine)	6.316 (1.071–37.23)	**0.042**	3.551 (0.334–37.706)	0.293
Assistantship duration	0.932 (0.357–2.434)	0.886	1.544 (0.532–4.48)	0.424
**Inadequate knowledge of common medications and their effects in TGD health (Q-5)**				
Age	1.025 (0.94–1.118)	0.575	1.044 (0.946–1.153)	0.39
Gender (Reference: Female)	0.897 (0.344–2.34)	0.825	0.887 (0.301–2.608)	0.827
Department (Reference: Family Medicine)	1.98 (0.647–6.063)	0.232	2.003 (0.598–6.708)	0.26
Assistantship duration	0.9 (0.548–1.476)	0.676	0.94 (0.568–1.556)	0.809
**Inadequate knowledge of common surgical procedures in TGD health (Q-6)**				
Age	1.002 (0.92–1.093)	0.956	0.99 (0.899–1.09)	0.841
Gender (Reference: Female)	0.942 (0.341–2.608)	0.909	0.742 (0.227–2.425)	0.621
Department (Reference: Family Medicine)	0.547 (0.192–1.554)	0.257	0.397 (0.121–1.304)	0.128
Assistantship duration	0.853 (0.509–1.429)	0.546	0.83 (0.487–1.413)	0.492
**Inadequate knowledge level regarding routine follow-up and screening for TGD individuals (Q-8)**				
Age	1.053 (0.958–1.157)	0.285	1.05 (0.945–1.165)	0.366
Gender (Reference: Female)	1.347 (0.514–3.528)	0.545	1.097 (0.37–3.251)	0.868
Department (Reference: Family Medicine)	0.96 (0.351–2.627)	0.937	0.995 (0.328–3.02)	0.993
Assistantship duration	0.82 (0.505–1.332)	0.422	0.818 (0.496–1.351)	0.433
**Inadequate knowledge level regarding routine follow-up and screening for TGD individuals (Q-9)**				
Age	0.92 (0.772–1.096)	0.35	0.93 (0.742–1.165)	0.527
Gender (Reference: Female)	0.487 (0.094–2.514)	0.39	0.803 (0.128–5.05)	0.815
Department (Reference: Family Medicine)	3.167 (0.822–12.192)	0.094	2.224 (0.461–10.722)	0.319
Assistantship duration	1.114 (0.558–2.227)	0.759	1.398 (0.637–3.069)	0.403
**Inadequate knowledge level regarding routine follow-up and screening for TGD individuals (Q-10)**				
Age	0.97 (0.894–1.052)	0.459	0.961 (0.873–1.057)	0.411
Gender (Reference: Female)	0.861 (0.34–2.178)	0.752	1.045 (0.366–2.983)	0.935
Department (Reference: Family Medicine)	1.451 (0.553–3.808)	0.449	1.043 (0.352–3.092)	0.939
Assistantship duration	1.054 (0.654–1.697)	0.829	1.138 (0.698–1.856)	0.603
**Inadequate knowledge level regarding routine follow-up and screening for TGD individuals (Q-11)**				
Age	0.994 (0.922–1.071)	0.874	0.975 (0.894–1.064)	0.575
Gender (Reference: Female)	1.605 (0.645–3.994)	0.309	2.079 (0.728–5.941)	0.172
Department (Reference: Family Medicine)	1.479 (0.564–3.877)	0.426	1.585 (0.534–4.703)	0.406
Assistantship duration	1.114 (0.694–1.789)	0.656	1.187 (0.729–1.932)	0.491
**Inadequate knowledge level regarding routine follow-up and screening for TGD individuals (Q-12)**				
Age	1.028 (0.952–1.11)	0.48	1.073 (0.977–1.178)	0.14
Gender (Reference: Female)	0.569 (0.206–1.578)	0.279	0.513 (0.152–1.738)	0.284
Department (Reference: Family Medicine)	3.136 (1.158–8.495)	**0.025**	2.653 (0.857–8.212)	0.091
Assistantship duration	0.686 (0.384–1.223)	0.201	0.753 (0.414–1.368)	0.351
**Inadequate knowledge level regarding routine follow-up and screening for TGD individuals Q-13)**				
Age	0.995 (0.887–1.115)	0.926	1.037 (0.921–1.167)	0.549
Gender (Reference: Female)	1.605 (0.645–3.994)	0.309	2.079 (0.728–5.941)	0.172
Department (Reference: Family Medicine)	4.024 (1.092–14.831)	**0.036**	4.876 (1.146–20.747)	**0.032**
Assistantship duration	1.014 (0.505–2.035)	0.97	1.16 (0.545–2.468)	0.7
**Inadequate knowledge level regarding routine follow-up and screening for TGD individuals (Q-14)**				
Age	0.89 (0.752–1.053)	0.175	0.952 (0.794–1.141)	0.594
Gender (Reference: Female)	0.122 (0.015–0.992)	**0.049**	0.157 (0.017–1.425)	0.1
Department (Reference: Family Medicine)	2.382 (0.724–7.836)	0.153	1.169 (0.307–4.447)	0.819
Assistantship duration	0.785 (0.381–1.614)	0.51	0.849 (0.375–1.922)	0.694
**Inadequate knowledge level regarding discrimination faced by TGD individuals in accessing healthcare services (Q-15)**				
Age	1.034 (0.928–1.152)	0.541	1.064 (0.941–1.203)	0.324
Gender (Reference: Female)	0.58 (0.109–3.079)	0.523	0.388 (0.055–2.718)	0.341
Department (Reference: Family Medicine)	0.889 (0.166–4.755)	0.891	0.952 (0.155–5.822)	0.957
Assistantship duration	1.044 (0.475–2.295)	0.915	1.02 (0.464–2.244)	0.96
**Inadequate knowledge level about the lack of access to health services among TGD individuals (Q-16)**				
Age	0.961 (0.867–1.064)	0.442	0.97 (0.861–1.092)	0.614
Gender (Reference: Female)	0.58 (0.185–1.816)	0.35	0.701 (0.196–2.499)	0.583
Department (Reference: Family Medicine)	1.5 (0.515–4.37)	0.457	1.171 (0.347–3.945)	0.799
Assistantship duration	0.918 (0.522–1.614)	0.766	0.945 (0.516–1.733)	0.856
**Lack of formal education on TGD health (Q-18)**				
Age	1.058 (0.965–1.161)	0.231	1.059 (0.948–1.183)	0.309
Gender (Reference: Female)	2.012 (0.756–5.356)	0.161	2.219 (0.706–6.969)	0.172
Department (Reference: Family Medicine)	2.046 (0.71–5.898)	0.185	2.457 (0.758–7.969)	0.134
Assistantship duration	0.57 (0.34–0.957)	**0.033**	0.576 (0.334–0.993)	**0.047**
**The inadequacy of formal education on TGD health (Q-20)**				
Age	1.044 (0.944–1.154)	0.403	1.029 (0.923–1.148)	0.604
Gender (Reference: Female)	2.012 (0.756–5.356)	0.161	2.219 (0.706–6.969)	0.172
Department (Reference: Family Medicine)	0.307 (0.036–2.6)	0.279	0.392 (0.043–3.564)	0.405
Assistantship duration	1.552 (0.817–2.947)	0.18	1.445 (0.767–2.721)	0.255
**The willingness to receive formal education on TGD health (Q-21)**				
Age	0.988 (0.885–1.103)	0.831	1.048 (0.91–1.206)	0.514
Gender (Reference: Female)	0.175 (0.021–1.454)	0.107	0.15 (0.014–1.624)	0.119
Department (Reference: Family Medicine)	2.773 (0.82–9.379)	0.101	1.59 (0.359–7.047)	0.541
Assistantship duration	0.738 (0.341–1.598)	0.441	0.813 (0.334–1.98)	0.649

Univariate: binary logistic regression; Multiple: multivariate logistic regression. Data are presented as odds ratio (OR) with 95% confidence interval (CI) and *p*-value. Bold values indicate statistical significance at *p* < 0.05.

## Data Availability

The data presented in this study are available on request from the corresponding author.

## References

[1] De Vries E., Kathard H., Müller A. (2020). Debate: Why Should Gender-Affirming Health Care Be Included in Health Science Curricula?. BMC Med. Educ..

[2] Oliphant J., Veale J., Macdonald J., Carroll R., Johnson R., Harte M., Stephenson C., Bullock J. (2018). Guidelines for Gender Affirming Healthcare for Gender Diverse and Transgender Children, Young People and Adults in Aotearoa New Zealand.

[3] American College of Obstetricians and Gynecologists’ Committee on Gynecologic Practice, Committee on Health Care (2021). Health Care for Transgender and Gender Diverse Individuals: ACOG Committee Opinion, Number 823. Obstet. Gynecol..

[4] Braun H.M., Garcia-Grossman I.R., Quiñones-Rivera A., Deutsch M.B. (2017). Outcome and Impact Evaluation of a Transgender Health Course for Health Profession Students. LGBT Health.

[5] Pharr J.R., Kachen A., Cross C. (2019). Health Disparities Among Sexual Gender Minority Women in the United States: A Population-Based Study. J. Community Health.

[6] de Vries J.M.A., Downes C., Sharek D., Doyle L., Murphy R., Begley T., McCann E., Sheerin F., Smyth S., Higgins A. (2023). An Exploration of Mental Distress in Transgender People in Ireland with Reference to Minority Stress and Dissonance Theory. Int. J. Transgender Health.

[7] Safer J.D., Coleman E., Feldman J., Garofalo R., Hembree W., Radix A., Sevelius J. (2016). Barriers to Healthcare for Transgender Individuals. Curr. Opin. Endocrinol. Diabetes Obes..

[8] Soled K.R.S., Dimant O.E., Tanguay J., Mukerjee R., Poteat T. (2022). Interdisciplinary Clinicians’ Attitudes, Challenges, and Success Strategies in Providing Care to Transgender People: A Qualitative Descriptive Study. BMC Health Serv. Res..

[9] Clark B.A., Veale J.F., Greyson D., Saewyc E. (2018). Primary Care Access and Foregone Care: A Survey of Transgender Adolescents and Young Adults. Fam. Pract..

[10] Vance S.R., Halpern-Felsher B.L., Rosenthal S.M. (2015). Health Care Providers’ Comfort with and Barriers to Care of Transgender Youth. J. Adolesc. Health.

[11] Unger C.A. (2015). Care of the Transgender Patient: A Survey of Gynecologists’ Current Knowledge and Practice. J. Womens Health.

[12] Coutin A., Wright S., Li C., Fung R. (2018). Missed Opportunities: Are Residents Prepared to Care for Transgender Patients? A Study of Family Medicine, Psychiatry, Endocrinology, and Urology Residents. Can. Med. Educ. J..

[13] Stupiansky N.W., Liau A., Rosenberger J., Rosenthal S.L., Tu W., Xiao S., Fontenot H., Zimet G.D. (2017). Young Men’s Disclosure of Same Sex Behaviors to Healthcare Providers and the Impact on Health: Results from a US National Sample of Young Men Who Have Sex with Men. AIDS Patient Care STDs.

[14] Furness B.W., Goldhammer H., Montalvo W., Gagnon K., Bifulco L., Lentine D., Anderson D. (2020). Transforming Primary Care for Lesbian, Gay, Bisexual, and Transgender People: A Collaborative Quality Improvement Initiative. Ann. Fam. Med..

[15] Click I.A., Mann A.K., Buda M., Rahimi-Saber A., Schultz A., Shelton K.M., Johnson L. (2020). Transgender Health Education for Medical Students. Clin. Teach..

[16] Leandre F., Diaz-Fernandez V., Ginory A. (2021). Are Medical Students and Residents Receiving an Appropriate Education on LGBTQ+ Health?. Aust. New Zealand J. Psychiatry.

[17] van Heesewijk J., Kent A., van de Grift T.C., Harleman A., Muntinga M. (2022). Transgender Health Content in Medical Education: A Theory-Guided Systematic Review of Current Training Practices and Implementation Barriers & Facilitators. Adv. Health Sci. Educ. Theory Pract..

[18] Obedin-Maliver J., Goldsmith E.S., Stewart L., White W., Tran E., Brenman S., Wells M., Fetterman D.M., Garcia G., Lunn M.R. (2011). Lesbian, Gay, Bisexual, and Transgender-Related Content in Undergraduate Medical Education. JAMA.

[19] Liang J.J., Gardner I.H., Walker J.A., Safer J.D. (2017). Observed Deficiencies in Medical Student Knowledge of Transgender and Intersex Health. Endocr. Pract..

[20] Vinekar K., Rush S.K., Chiang S., Schiff M.A. (2019). Educating Obstetrics and Gynecology Residents on Transgender Patients: A Survey of Program Directors. Obstet. Gynecol..

[21] Foglia L.M., Gilmandyar D., Jimenez E.A., Lee Shanks A.I., Tache V., McLeod F.N., Hechanova M.L., Scott Schellhammer S., George K.E., Chair C. (2022). Council on Resident Education in Obstetrics and Gynecology (CREOG) Educational Objectives: Core Curriculum in Obstetrics and Gynecology. https://www.acog.org/-/media/project/acog/acogorg/files/creog/creog-educational-objectives-13th-edition.pdf?rev=53bf30ec0f144132a8d81d81549b2476.

[22] Grimstad F.W., Satterwhite C.L., Wieneke C.L. (2016). Assessing Residency Program Approaches to the Transgender Health CREOG Objective. Transgender Health.

[23] Donovan M., Vanderkolk K., Graves L., McKinney V.R., Everard K.M. (2021). Gender-Affirming Care Curriculum in Family Medicine Residencies: A CERA Study. Fam. Med..

[24] Vanderkolk K., McKinney V.R., Graves L., Harper D.M. (2021). Transgender Education in North American Family Medicine Clerkships: A Cera Study. Fam. Med..

[25] Pinto A.D., Glattstein-Young G., Mohamed A., Bloch G., Leung F.H., Glazier R.H. (2016). Building a Foundation to Reduce Health Inequities: Routine Collection of Sociodemographic Data in Primary Care. J. Am. Board Fam. Med..

[26] Safer J.D., Tangpricha V. (2019). Care of the Transgender Patient. Ann. Intern. Med..

[27] Coleman E., Radix A.E., Bouman W.P., Brown G.R., de Vries A.L.C., Deutsch M.B., Ettner R., Fraser L., Goodman M., Green J. (2022). Standards of Care for the Health of Transgender and Gender Diverse People, Version 8. Int. J. Transgender Health.

[28] Maschião L.F., Bastos F.I., Wilson E., Mcfarland W., Turner C., Pestana T., Veras M.A. (2020). Nonprescribed Sex Hormone Use Among Trans Women: The Complex Interplay of Public Policies, Social Context, and Discrimination. Transgender Health.

[29] Asscheman H., Giltay E.J., Megens J.A.J., De Ronde W., Van Trotsenburg M.A.A., Gooren L.J.G. (2011). A Long-Term Follow-up Study of Mortality in Transsexuals Receiving Treatment with Cross-Sex Hormones. Eur. J. Endocrinol..

[30] Sánchez-Toscano E., Domínguez-Riscart J., Larrán-Escandón L., Mateo-Gavira I., Aguilar-Diosdado M. (2023). Cardiovascular Risk Factors in Transgender People after Gender-Affirming Hormone Therapy. J. Clin. Med..

[31] Rufino A.C., Madeiro A., Trinidad A., Santos R., Freitas I. (2018). Sexual Practices and Health Care of Women Who Have Sex with Women: 2013–2014. Epidemiol. Serv. Saude.

[32] Sabin J.A., Riskind R.G., Nosek B.A. (2015). Health Care Providers’ Implicit and Explicit Attitudes Toward Lesbian Women and Gay Men. Am. J. Public Health.

[33] Ettner R., Monstrey S., Coleman E. (2016). Principles of Transgender Medicine and Surgery.

[34] Boehmer U., Gereige J., Winter M., Ozonoff A. (2019). Cancer Survivors’ Access to Care and Quality of Life: Do Sexual Minorities Fare Worse than Heterosexuals?. Cancer.

[35] Stenzel A.E., Bustamante G., Sarkin C.A., Harripersaud K., Jewett P., Teoh D., Vogel R.I. (2022). The Intersection of Sexual Orientation with Race and Ethnicity in Cervical Cancer Screening. Cancer.

[36] Eckhert E., Lansinger O., Ritter V., Liu M., Han S., Schapira L., John E.M., Gomez S., Sledge G., Kurian A.W. (2023). Breast Cancer Diagnosis, Treatment, and Outcomes of Patients from Sex and Gender Minority Groups. JAMA Oncol..

[37] Walensky R.P., Jernigan D.B., Bunnell R., Layden J., Kent C.K., Gottardy A.J., Leahy M.A., Martinroe J.C., Spriggs S.R., Yang T. (2021). Sexually Transmitted Infections Treatment Guidelines, 2021. MMWR Recomm. Rep..

